# It Is Complicated: The Medico-Social Journey of an Undocumented Pregnant Adolescent

**DOI:** 10.1155/2020/6749630

**Published:** 2020-04-28

**Authors:** Helen Kest, Ashlesha Kaushik, Bushra Tehreem, David Goldberg

**Affiliations:** ^1^Department of Pediatrics, St. Joseph's Health, 703 Main Street, Paterson, NJ 07503, USA; ^2^Unity Point Health, Siouxland Medical Education Foundation and University of Iowa Carver College of Medicine, 2720 Stone Park Blvd, Sioux City, IA 51104, USA

## Abstract

Adolescent pregnancies are a global health problem with over 16 million children born to this age group globally. Adolescent females also represent almost half of all adolescent global migrants. Adolescent pregnancy by itself is associated with poor health care access and morbidities; the additional risk of social insecurity in the case of undocumented adolescent migrants leads to higher risks for the mother and newborn. According to the CDC, adolescents comprise half of all new sexually transmitted diseases (STDs) including reported primary and secondary syphilis. Our case highlights the relationship between social insecurity for the undocumented adolescent migrant and excess risks for preventable mother-to-child transmission of communicable diseases. In formulating preventive measures and policies for the recent rise in sexually transmitted maternal diseases with resultant congenital infections, there is need for health care systems and providers to familiarize themselves with advocacy and other useful resources that will promote health care access for undocumented and other vulnerable adolescents. Additionally, local providers who work in areas with a large population of immigrant adolescents should utilize the untapped resources of these adolescents to develop youth community advocacy projects that link adolescents to health resources, including reproductive health.

## 1. Introduction

Social insecurity, low income, and adolescent pregnancy are known perinatal risk factors that independently increase the risk of maternal, perinatal, and neonatal morbidity. Adolescent pregnancies (15–19-year-old) are a global health problem associated with poor health and social outcomes and more likely to occur in marginalized/vulnerable communities. About 16 million children are born to this age group globally [1].

We present a case of congenital syphilis, in an adolescent pregnant teen who did not seek prenatal care due to undocumented status resulting in undiagnosed, untreated syphilis and congenital neurosyphilis in her newborn. Through this unique case, we present a review of the rising trend of congenital syphilis, its presentation, and complications.

We also review the current state of health care policies and access for undocumented pregnant adolescents. In our conclusion, we propose the need for our diverse collective community as partner-advocates to familiarize ourselves with available resources for health access in undocumented persons and to engage the untapped resources of adolescent stakeholders in developing awareness and integration programs that will promote access for vulnerable populations of other adolescents.

## 2. Case Report

A 17-year-old primigravida from Latin America, who did not seek care during pregnancy reportedly due to her undocumented status and fear of consequences, presented to a local community hospital in labor. Duration of rupture of membranes (ROM) was unknown. Her RPR (Rapid Plasma Reagin) titer was reported as >1 : 64. Due to heavy meconium, the baby was delivered by emergency C-section. A female neonate weighing 2.81 kg was born at 37.3 weeks of gestation. The baby was hemodynamically stable and had Apgar scores of 9/9 at 1 and 5 minutes, respectively. However, the baby was noted to have persistent hypoglycemia despite regular feeds and was admitted to a level II NICU where blood work showed a RPR titer of >1 : 64, platelet count of 69 × 109/L, and cholestasis with direct hyperbilirubinemia. The patient was transferred to our hospital for level 3 NICU care and for further workup and treatment. Examination showed that the patient was jaundiced, lethargic, and tachypenic, and in mild respiratory distress (RR was 72/min). Physiologic reflexes and muscle tone were normal. There were no clinical features of congenital infection on examination.

Laboratory tests showed hypoglycemia (46 mg/dl), thrombocytopenia (69 × 109/L), and normal counts of leucocytes and neutrophils. Blood chemistry showed a total bilirubin of 11.4, with direct bilirubin of 7.00, indirect bilirubin of 4.4, and elevated liver enzymes (ALT/AST of 185/79 unit/L). CSF studies showed elevated leukocytes of 30/mm3, neutrophils of 46%, and elevated proteins of 106 mg/dl. No microorganism was found in CSF culture. CSF VDRL was 1 : 8 (reference range = <1.1), indicating the diagnosis of neurosyphilis. Testing for HIV, Rubella, CMV, and toxoplasma was negative. Blood HSV 1 and 2 PCR was negative. Nontreponemal serologic screen (RPR) was reactive with high titers of 1: 256. FTA-ABS was reactive.

The infant's long bone X-rays showed signs of syphilitic osteochondritis in all bones with zones of lucency seen bilaterally in proximal and distal ends of tibia and fibula, proximal humeral metaphysis, and proximal and distal femoral metaphysis suggestive of congenital syphilis infection. Abdominal ultrasound showed mild prominence of spleen for age with mild perihepatic free fluid. Chest radiograph showed absence of focal infiltrates, and no osseous changes were noted. Ultrasound of the brain revealed no specific pathology. Auditory examination using the optoacoustic emission test was normal. Ophthalmologic examination revealed no pathology. Intravenous penicillin G 50,000 u/kg/dose every 12 h for 7 days was started and switched to every 8 hours to complete a 10-day course. The clinical course remained stable throughout treatment. After starting the treatment, gradual improvement was noticed in platelet count. Hepatic cholestasis resolved and jaundiced improved.

The mother was also treated with three doses of long-acting Benzathine penicillin G (2.4 million units administered intramuscularly) at weekly intervals. The patient was discharged home with the mother after 18 days stay in the NICU. The infant was clinically stable and feeding actively. The following recommendations were given on discharge: monitoring by general pediatrician, follow-up with an infectious disease specialist, and repeat serologic testing at 3 months.

Ancillary support including social services was provided for education of available community and other resources and continued medical care at the time of discharge.

## 3. Discussion

### 3.1. Congenital Syphilis (CS)

CS is caused by bacteria *Treponema pallidum* through vertical transmission. *T. pallidum* is a small, spiral-shaped, venereal, disease-causing treponeme. Humans are the only known natural host. *T. pallidum* can cross the placenta at any stage of pregnancy, and transmission of infection is independent of the stage of maternal disease [2]. The risk of fetal infection is directly related to gestational age.

Like adult syphilis, congenital syphilis is divided into stages: early CS manifests in the first 2 years of life and late CS manifests after 2 years of life. Early CS can present as early as during gestation with serious outcomes as still birth, early fetal loss, hydrops fetalis, or premature birth. Babies born at term can have variable presentation. Two thirds of the babies with CS are asymptomatic at birth [2, 3]. Untreated and asymptomatic neonates display symptoms by 5–15 weeks of age. Manifestations can involve mucocutaneous, reticuloendothelial, neurological, ocular, and other organ involvement. Mucocutaneous infection is seen in 70% of infected infants and may present immediately at birth or few weeks later. The most common cutaneous manifestation is small dark red copper spots, most obvious on hands and feet. Bone involvement frequently seen in untreated CS includes periostitis (as seen in our patient) involving metaphyseal and diaphyseal portions of long bone or osteochondritis involving joints, and pain and pseudo-paralysis in the affected limbs. Other manifestations are rhinitis, snuffles, nontender lymphadenopathy, hepatosplenomegaly, hemolytic anemia, thrombocytopenia, leukopenia, leukocytosis, osteochondritis, neurosyphilis, or pneumonia [3].

Late congenital syphilis is a rare presentation in developed countries. Untreated infants with no manifestations at birth or early infancy can present with late CS having CNS, bones, eyes, teeth, and skin involvement as late as 2 years. Hutchinson's triad may not become apparent until many years later. The triad includes interstitial keratitis, cranial nerve eight deafness, and notched teeth called Hutchinson's teeth. CNS manifestations include mental retardation, hydrocephalous, seizure disorder, saddle nose, cluttons joints, high palatal vault, frontal bossing, and other signs.

Diagnosis of CS requires both nontreponemal and treponemal serologic testing. Nontreponemal tests include VDRL and RPR. The reactive nontreponemal test should always be confirmed with the treponemal test to exclude false positive test results. Treponemal testing includes *T. pallidum* particle agglutination test, TP-EIA, and TP-FTAS. Neurosyphilis can be asymptomatic and is diagnosed on the basis of CSF studies with high WBC count (>WBC/mm3) and high CSF proteins (>40 mg/dl) and reactive CSF VDRL results as seen in our patient. Nonreactive CSF VDRL does not exclude the diagnosis of neurosyphilis due to poor sensitivity during the neonatal period [4]. CSF FTA-ABS can support the diagnosis but cannot help establishing the diagnosis of congenital syphilis. X-rays can show lucencies or sclerotic bone changes. Evaluation and treatment of congenital syphilis depends on possible, probable, or confirmed congenital syphilis; parenteral penicillin G remains the drug of choice for treatment.

All women should be screened serologically for syphilis early in pregnancy. Pregnant women with reactive treponemal screening tests should have additional quantitative nontreponemal testing, because titers are essential for monitoring treatment response. For communities and populations in which the prevalence of syphilis is high and for women at high risk for infection, serologic testing should also be performed twice during the third trimester: once at 28–32 weeks gestation and again at delivery. Any woman who has a fetal death after 20 weeks gestation should be tested for syphilis. Penicillin G is the only known effective antimicrobial for preventing maternal transmission to the fetus and treating fetal infection.

The Centers for Disease Control and Prevention (CDC) recommends one of two regimens for treatment of an infant born to a woman with any stage of syphilis who has not received appropriate treatment during gestation or for whom the possibility of congenital syphilis cannot be ruled out: Aqueous crystalline penicillin G 100,000–150,000 units/kg/day is administered intravenously every 12 hours at a dose of 50,000 units/kg/dose during the first seven days of life and every eight hours thereafter, for a total of 10 days, or Procaine penicillin G 50,000 units/kg/dose intramuscular (IM) is administered in a single daily dose for 10 days up to the adult maximum dose of 2.4 million units/dose.

### 3.2. Social and Ethical Issues Surrounding Undocumented Migration

According to the United Nations (UN) Global Migration Group, migrants between 15 and 24 years of age represented about one-eighth (28.2 million) of the 232 million international migrants worldwide in 2013. Forty-six percent of migrants aged 15–24 are young adolescent women or girls [5].

The United States (US) has the largest foreign-born population in the world, continues to be the largest country-to-country migration corridor in the entire world, and has been the main country of destination for international migrants since 1970 [4].

The combination of these and other factors has led to many fierce and deeply dividing political and policy debates that center on undocumented immigrants. This may in part explain poor health care access with the potential consequence for poor health outcomes. Accordingly, the socially disadvantaged position of immigrants leads to negative emotions and subsequent changes in health behaviors, including the utilization of health care [6].

Migrant adolescents often undergo dangerous journeys to escape situations in their homeland travelling far in hopes of finding a new world full of possibilities. Yet on arrival, most must face the harsh reality of lack of access to essential services including health care. Even in many developed countries where these services are protected by well-intended policies, these youth migrants must overcome other obstacles driven largely by fear of apprehension, financial and linguistic barriers, and the risk that using even essential services will reveal their status and result in detention or deportation [6, 7].

### 3.3. Adolescent Pregnancy and Risks for Congenital Syphilis

Adolescent pregnancy by itself is associated with poor health care access and morbidities; the additional risk of social insecurity in case of the undocumented adolescent leads to higher risks for the mother and newborn. According to the CDC, in the US, adolescents (15–24 years) comprise half of all new sexually transmitted diseases (STDs), and during 2015–2016, the rate of reported primary and secondary syphilis cases increased 13.0% among persons aged 15–19 years [8]. There was also a concomitant increase in cases of congenital syphilis cases during this period. In 2016, there were 628 reported cases of congenital syphilis (CS), including 41 stillbirths. According to the CDC, reported cases of congenital syphilis showed an increase from 362 in 2013 to 918 in 2017, which is the highest number in 20 years. Rates are highest among minorities including blacks and Hispanics.

CS is a preventable disease that can be treated during pregnancy with follow-up serologies to determine disease status. A systemic review by Quin et al. [9] to estimate the probability of adverse pregnancy outcomes (APOs) among women with and without syphilis showed absolute differences between syphilitic mothers and nonsyphilitic mothers for preterm birth, low birth weight, stillbirth or fetal loss, miscarriage, and neonatal death. For syphilitic women with high titers as in this case, APOs were at significantly higher proportions at 29%, 8%, 4%, 13%, and 14%, respectively.

Due to its preventable nature, any case of CS is one too many. CS creates a huge global burden in terms of complications for the mother and child. The global burden of mother-to-child transmission (MTCT) of syphilis is estimated at 3.6 million disability-adjusted life years (DALYs), comparable to MTCT of human immunodeficiency virus and around $309 million in medical costs [10, 11].

Studies have shown direct relationship between undocumented migrant status and poor access to health services [12] with excess risks for communicable disease-related morbidity and costs [13]. Perhaps, “related” includes the prevalence data from population-based surveys that indicate marked disparities in minority populations for both reportable and nonnationally reportable STDs [14].

Studies analyzing mathematical models for STDs addressing important epidemiological implications have been reported [15–19]. Mathematical models could provide additional insights in disease epidemiology with the ultimate goal of formulating preventive measures for halting the rise in sexually transmitted infections.

The recent increase of congenital syphilis in the US in vulnerable populations is a strong call for advocacy and promotion of policies that targets improved access for earlier detection and treatment of maternal infection to avoid adverse outcomes in neonates.

### 3.4. Policy, Immigrant Health Access, and the Role of the Health Care Provider

The ongoing debates on healthcare coverage for undocumented persons are complex with philosophical, political, and ethical implications due to issues relating to type and scope of medical services and the consequent cost burden on taxpayers. Physicians are at first individuals with varied opinions and preferences that are integral to their own self-conceptions or identity. However, with regards to healthcare, most healthcare providers agree that the professional code of ethics should promote nondiscrimination and are obliged to establish relationships that are “not necessarily binding for the rest of the population or for the same persons outside of that relationship” [20].

Congenital syphilis in minorities continues to be an important contributor to disparities in mother-to-child transmission (MTCT) of congenital diseases resulting in substantial perinatal morbidity, making its elimination a public health priority.

Health care providers should familiarize themselves with available resources for immigrant teens. The American Academy of Pediatrics' (AAP) advocacy and leadership support for immigrant health are instrumental in playing a pivotal role in ensuring continued support for health of all children irrespective of their backgrounds. The AAP Immigrant Child Health toolkit contains information and links to useful resources [21]. It also contains important information on advocacy opportunities.

Additionally, local providers who work in areas with a large population of immigrant adolescents should utilize the untapped resources of these adolescents to develop youth community advocacy projects that link adolescents to health resources, including reproductive health. Preventing adverse neonatal outcomes is an ethical responsibility shared by all providers that resonates with the AAP's mission.

## 4. Conclusion

The rising trend of CS, a largely preventable disease, in the US can be explained by poor health access during pregnancy. Several factors affect health care access, but a mostly understudied area is the social and economic determinants in undocumented teens and youths. Health care systems and providers should familiarize themselves with advocacy and other useful resources to combat this trend.
